# Modulating effect of *Gmelina arborea* Linn. on immunosuppressed albino rats

**DOI:** 10.4103/0974-8490.75455

**Published:** 2010

**Authors:** S. H. Shukla, A. K. Saluja, S. S. Pandya

**Affiliations:** * *Indukaka Ipcowala College of Pharmacy, New Vallabh Vidyanagar, Dist. Anand, Gujarat - 388 121, India*; 1 *A.R.College of Pharmacy, Vallabh Vidyanagar, Dist. Anand, Gujarat - 388 120, India*; 2 *Nutan Education Trust’s B. Pharmacy College, At. Rampura, Po. Kakanpur, Tal. Godhra, Dist. Panchmahal, Gujarat - 389 713, India*

**Keywords:** Cellular immunity, *Gmelina arborea*, Humoral immunity, Immunomodulation

## Abstract

**Aim:**

In the present study, the immunomodulatory effects of roots of *Gmelina arborea* Linn. were investigated

**Materials and Methods:**

Methanolic extract of *G. arborea* Linn. (MEGA) and its ethyl acetate fraction (EAFME) were used for evaluating the pharmacological activity. The modulating effect was evaluated on humoral and cell-mediated immune response using animal models like cyclophosphamide-induced myelosuppression, delayed-type hypersensitivity (DTH) response, and humoral antibody (HA) titre

**Results:**

Both test extracts produced significant increase in HA titre, DTH response, and levels of total white blood cell count

**Conclusion:**

This drug is found to be a potential immunostimulant

## INTRODUCTION

Medicinal plants have been used to cure human illness since time immemorial. Certain of these drugs are believed to promote positive health and maintain organic resistance against infections by re-establishing body equilibrium and conditioning the body tissue.[1,2]

The Indian System of Medicine “Ayurveda” conceptualizes a category of drug activity known as “Rasayana”. The word “Rasayana” is composed of two words “Rasa” meaning elixir and “ayana” meaning house. The word, therefore, signifies property of the plant that helps to rejuvenate the system.[3] Many plants have been extensively used as “Rasayana” drugs in Ayurveda for the management of neurodegenerative diseases, as rejuvenators, immunomodulators, aphrodisiac, and nutritional supplements.[4,5]

The root of *G. arborea* Linn. is one of the ingredients of “*dashmuladikwath*” and “*bhrihatpanchamool*” of ayurveda, which constitutes a number of ayurvedic preparations used as tonics.[6] According to the Ayurvedic literature, the roots of *G. arborea* Linn. have been reported to be used in case of hallucination, fever, dyspepsia, hyperdipsia, hemorrhoids, gastralgia, anasarca, and in burning sensation. It is bitter, sweet, tonic, laxative, galactogogue, and anthelmintic.[7]

It was considered worthwhile to investigate this drug for its effect on humoral and cell-mediated immunity in normal as well as cyclophosphamide-induced myelosuppression in rats

## MATERIALS AND METHODS

### Animals

Wistar albino rats of either sex weighing 150−300 g were used and housed in 12-h light/12-h dark cycles, under temperature controlled (20 ± 2°C) conditions, and relative humidity of 50−55%. The animals were fed with standard pellet diet and water *ad libitum*. Animal experiments were approved by the Institutional Animal Ethics Committee constituted as per the directions of the Committee for the Purpose of Control and Supervision of Experimental Animals, Chennai, India

#### Collection and preparation of plant material

The roots were collected from Nadiad District of Gujarat, India and authenticated by Pharmacognosist of Indukaka Ipcowala College of Pharmacy, New Vallabh Vidyanagar. A voucher specimen (IICP/06/01) has been preserved in our laboratory. The collected plant material was cut into small pieces and dried under shade. The material was then powdered (# 60) with mechanical grinder and stored in air tight container.

### Preparation of extracts

The dry powdered material was defatted using petroleum ether (60−80°) using Soxhlet’s extractor. Defatted material was then extracted with chloroform followed by methanol and finally with water. The solvents were completely removed under reduced pressure. The methanolic and aqueous extract obtained as a semisolid mass were selected for further studies after preliminary phytochemical analysis of all extracts.[8] After pharmacological screening, fractionation of the extract showing significant pharmacological activity was carried out. Thus, methanolic extract was fractionated using solvents like chloroform and ethyl acetate.

Aqueous extract did not show significant pharmacological activity, thus its fractionation was not carried out

Finally, pharmacological activity of defatted methanolic extract of *Gmelina arborea* (MEGA) and ethyl acetate fraction of methanolic extract (EAFME) was carried out

### Selection of dose

Five different dose levels (50−600 mg/kg body weight) of both the extracts were taken for selection of doses. After screening five different dose levels, statistical test was applied and the doses showing significant results were chosen. Two dose levels, i.e., 300 and 500 mg/kg for MEGA; 50 and 100 mg/kg for EAFME were selected and finally screened for pharmacological activity. Aqueous extract did not show any pharmacological activity upto 800 mg/kg dose level. Toxicity studies of roots of *G. arborea* was carried out earlier[9] and as per the suggestions of the Instituitional Animal Ethics Committee (IAEC) members it was not repeated

### Drugs

Accurately weighed quantities of test extracts were triturated with water. Cyclophosphamide was used as a standard immunosuppressant drug. Sheep Red Blood Cells (SRBCs) were used as an antigen at concentration of 20% (5 × 10^9^ SRBCs/rat) for immunization and 1% (0.25 × 10^9^ SRBCs / rat) for challenge.

### Methods

#### Cyclophosphamide-induced myelosuppression

The method described by Manjrekar *et al*. (2000) was adopted.[10] Animals were divided into six groups of six animals each.

Group I (Normal control group) and Group II (Cyclophosphamide-treated group) received the vehicle (water) for period of 13 days. Groups III, IV, V, and VI were given dose of MEGA 300 mg/kg, MEGA 500 mg/kg, EAFME 50 mg/kg, and EAFME 100 mg/kg respectively, p.o., daily for 13 days. The animals of groups II-VI were injected with cyclophosphamide (30 mg/kg, i.p.) on the 11^th^, 12^th^, and 13^th^ day, 1 h after the administration of the respective drug treatments. Blood samples were collected from retro orbital plexus on the day before (day 0) and on the 14^th^ day of the experiment. Determination of total and differential white blood cells was carried out

#### Humoral Antibody (HA) and Delayed Type Hypersensitivity (DTH) response using SRBC as antigen

The method described by Puri *et al*. (1994) was adopted.[11] Group I (Normal control group) and Group II (Cyclophosphamide-treated group) received the vehicle (water) for a period of 7 days. Groups III, IV, V, and VI were given oral dose of MEGA 300 mg/kg, MEGA 500 mg/kg, EAFME 50 mg/kg, and EAFME 100 mg/kg respectively, daily for 7 days. The animals of groups II-VI were injected with cyclophosphamide (30 mg/kg, i.p.) on the 4^th^, 5^th^, and 6^th^ day, 1 h after the administration of the respective drug treatments

The animals were immunized by injecting 0.1 ml of 20% of fresh SRBC suspension, intraperitonially on day 0. Blood samples were collected in microcentrifuge tubes from individual animal from retro-orbital plexus on the 7^th^ day and serum was separated. Antibody levels were determined by hemagglutination technique. Briefly, equal volumes of individual serum samples of each group were pooled. Two fold dilutions of pooled serum samples were made in 25 μl volumes of normal saline in microtitration plate and to that was added 25μl of 1% suspension of SRBC in saline. After mixing, the plates were incubated at room temperature for 1 h and examined for hemagglutination under microscope. The reciprocal of the highest dilution of the test serum giving agglutination was taken as the antibody titre

The thickness of the right hind footpad was measured using vernier caliper on the 7^th^ day. The animals were then challenged by injecting 20μl of 1% SRBC in right hind foot pad and after 24 h and 48 h of this challenge the foot thickness was measured again. The pre-and post-challenge difference in the thickness of footpad was expressed in mm and taken as a measure of DTH

#### Statistical analysis

Data were expressed as mean ± S.E.M and statistical analysis was carried out using one way ANOVA followed by Dunnet’s multiple comparison tests using GraphPad InStat software

## RESULTS

### Preliminary phytochemical screening

**Figure 1:**
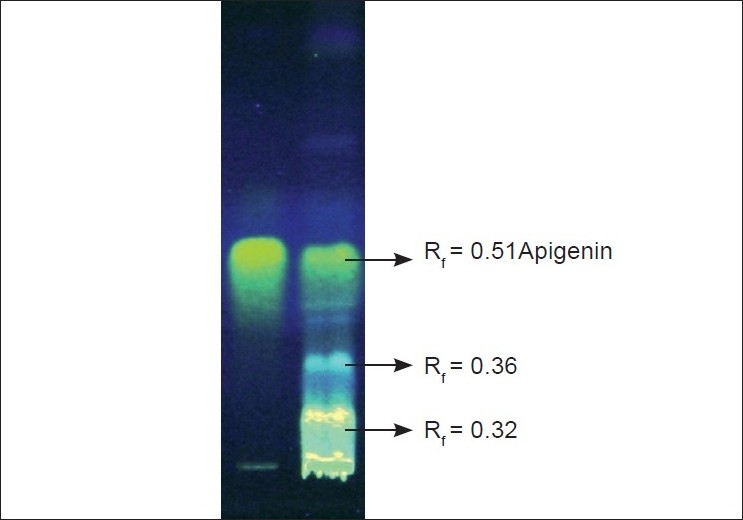
Co-TLC of Standard (STD)-apigenin and MEGA showing three fluorescent spots when observed at 254 nm after derivatisation with NP-PEG reagent.

Qualititative phytochemical screening of the defatted chloroform, methanol, and aqueous extract was carried out by reported methods.[12] MEGA showed the presence of flavonoids and lignans; aqueous extract showed the presence of lignans and carbohydrates, whereas EAFME showed only the presence of flavonoids. The presence of the constituents was then confirmed by thin layer chromatography (TLC) by reported methods.[13] Co-TLC of MEGA with standard confirmed the presence of apigenin, a flavonoid. It was done using precoated silica gel G F_254_ (E.Merck) plate as stationary phase; toluene: ethyl acetate: methanol (7:3:1, v/v) as mobile phase and Natural Product: PEG reagent as detecting reagent. The plate was observed at 254 nm under U.V. light. Three fluorescent spots of bright light blue, light blue, and yellowish green were observed having R_f_ value 0.32, 0.36, and 0.51 respectively. The spot having Rf = 0.51 corresponded with the spot of standard-apigenin [Figure 1]

### Cyclophosphamide-induced myelosuppression

A significant (*P* < 0.01) reduction in total white blood cell count was observed in rats treated with cyclophosphamide alone (group II) as compared to control group (group I). MEGA and EAFME increased the levels of total WBC count as compared to cyclophosphamide treated group. The rise in the total WBC count lowered by cyclophosphamide was observed at 500 mg/kg of MEGA, 50 mg/kg, and 100mg/kg of EAFME. The total WBC count was restored back to normal [Table 1].

**Table 1:** Effect of ME and EAFME of *Gmelina arborea* Linn. on Total WBC Count

Group	Treatment	Dose (mg/kg)	Total WBC (mm^3^)Mean ± S.E.M
I	Vehicle	-	9450.00 ± 301.94
II	CP	30	2583.33 ± 592.97**^a^
III	MEGA+CP	300	2766.67 ± 438.69#^b^
IV	MEGA+CP	500	9750.00 ± 387.08**^b^
V	EAFME+CP	50	6300.00 ± 118.32**^b^
VI	EAFME+CP	100	9200.00 ± 238.05**^b^

N = 6 rats per group,

# *P* > 0.05-insignificant

** *P* < 0.01;

a Group II was compared with group I,

b Groups III-VI were compared with group II

There was a significant (*P* < 0.01) decrease in Neutrophils and increase in lymphocytes in animals treated with cyclophosphamide (group II) as compared to control group (group I). MEGA at 500 mg/kg dose significantly (*P* < 0.01) increased the neutrophils as compared to group I, but failed to significantly reduce the lymphocyte count as compared to group II. EAFME normalized the neutrophil and lymphocyte count, which was lowered by cyclophosphamide [Table 2].

**Table 2:** Effect of ME and EAFME of *Gmelina arborea* Linn. on Differential WBC Count

Group	Treatment	Dose (mg/kg)	N	L	E	M	B
I	Vehicle	-	66.17 ± 1.58	30.17 ± 1.62	1.83 ± 0.31	1.83 ± 0.40	0
II	CP	30	53 ±2.48**^a^	44.17 ± 2.65**^a^	1.33 ± 0.21	1.5 ± 0.22	0
III	MEGA	300	51.83 ± 3.17#^b^	43.5 ± 3.98#^b^	1.5 ± 0.22	1.5 ± 0.34	0
IV	MEGA	500	62.33 ± 0.99**^b^	36.33 ± 2.23#^b^	1.5 ± 0.22	1.5 ± 0.22	0
V	EAFME	50	60.5 ± 1.18#^b^	36.33 ± 2.23#^b^	1.5 ± 0.22	1.5 ± 0.22	0
VI	EAFME	100	65.67 ± 0.72**^b^	31.5 ± 0.96**^b^	1.33 ± 0.21	1.5 ± 0.22	0

N - neutrophils, L- lymphocytes, E-erythrocytes, M- monocytes, B- basophils; N = 6 rats per group,

# *P* > 0.05-insignificant

** *P* < 0.01;

a Group II was compared with group I,

b Groups III-VI were compared with group II

### Effect of extracts on HA titre and DTH using SRBC as an antigen in rats

The animals treated with cyclophosphamide alone (group II) showed significant (*P* < 0.01) reduction in hemagglutinating antibody titre as compared to control animals (group I).The animals treated with MEGA at 300 mg/kg (*P* < 0.05) and 500 mg/kg (*P* < 0.01) showed a significant increase in HA titre as compared to cyclophosphamide treated animals (group II). EAFME showed a significant (*P* < 0.01) increase in HA titre as compared to animals treated with cyclophosphamide (group II).

The animals treated with cyclophosphamide and extracts showed a significant change in DTH response as compared to control animals (group I). As can be evident from Table 3, a significant (*P* < 0.01) increase in DTH response was observed at all levels of both the extracts as compared to cyclophosphamide-treated animals (group II).

**Table 3:** Effect of ME and EAFME of *Gmelina arborea* Linn. on HA titre and DTH response

Group	Treatment	Dose (mg/kg)	HA titre Mean ± S.E.M.	DTH response (mm) Mean paw edema ± S.E.M
I	Vehicle	-	191.14 ± 45.69	0.39 ± 0.013
II	CP	30	1.92 ± 0.29**^a^	1.27 ± 0.18**^a^
III	MEGA	300	122.88 ± 18.32*^b^	1.61 ± 0.01*^b^**^a^
IV	MEGA	500	191.15 ± 27.31**^b^	1.95 ± 0.04**^b^^a^
V	EAFME	50	47.79 ± 6.83#^b^	1.92 ± 0.05*^b^^a^
VI	EAFME	100	245.76 ± 36.64*^b^	2.37 ± 0.08*^b^^a^

N = 6 rats per group,

# *P* > 0.05-insignificant

* *P* > 0.05,

** *P* < 0.01;

a Group II was compared with group I,

b Groups III-VI were compared with group II

## DISCUSSION

A high degree of cell proliferation renders the bone marrow a sensitive target particularly to cytotoxic drugs. Loss of stem cells and inability of the bone marrow to regenerate new blood cells results in thrombocytopenia and leucopenia.[14]

Administration of MEGA and EAFME of *G. arborea* Linn. were found to increase the total WBC count, which was lowered by cyclophosphamide, a cytotoxic drug. The drug is also capable of normalising the levels of neutrophils and lymphocytes. The results of the present study indicate that the test drug can stimulate the bone marrow activity. As the drug is capable of reducing the cyclophosphamide induced toxicity, it can be useful in cancer therapy also

Antibody molecules, a product of B-lymphocytes and plasma cells, are central to humoral immune response; IgG and IgM are the major immunoglobulins which are involved in the complement activation, opsonization, neutralization of toxin etc.[15] The stimulation of the humoral response against SRBC by MEGA and EAFME as evidenced by the increase in HA titre in rats also indicate the enhanced responsiveness of macrophages and subsets of T and B lymphocytes, involved in antibody synthesis.[16]

Delayed type hypersensitivity is a well defined invivo model of cell-mediated response. DTH reaction can be quantified by measuring the paw thickness after injection of antigen.[17–19] SRBC injection increased the paw thickness within 18−24 h. The interaction of sensitized T-cell with presented antigen is known to be associated with the release of mediators such as histamine, products of arachidonic acid metabolism (prostaglandin or leukotrienes) and eventually interferon-γ leading to DTH.[20]

Animals treated with cyclophosphamide showed potentiation of DTH response as cyclophosphamide damaged the short lived suppressor cells in immune regulatory systems.[21] Increase in the DTH response indicates that *G. arborea* Linn. possesses stimulatory effect on lymphocytes and on other necessary cell types required for the expression of the reaction and also influence on biological mediators.[22,23]

The present study revealed that the plant possesses immunostimulatory effect, which may be due to the cell mediated activation of T and B cells. The plant shows the presence of flavonoids (apigenin), which is reported to exhibit immunomodulatory activity in various experimental models.[24,25] The presence of flavonoids in the plant might be contributing towards the modulating effect in immunosuppressed rats.

Further, present study supports the claims made in Ayurveda regarding the use of the roots of *G. arborea* Linn. as rasayana drugs under the common name of “gambhari”
